# Epigenome-wide exploratory study of blood DNA methylation and markers of physical performance indicates subtle differences across levels of cardiovascular fitness

**DOI:** 10.3389/fphys.2026.1713760

**Published:** 2026-04-15

**Authors:** Ferenc Torma, Emerson Santos, Mátyás Jókai, Zsófia Bábszky, Gergely Bábszky, Soroosh Mozaffaritabar, Roberto H. Herai, Ricardo A. Pinho, Mitsuru Higuchi, Yaodong Gu, Zsolt Radák

**Affiliations:** 1 Research Center for Molecular Exercise Science, Hungarian University of Sports Science, Budapest, Hungary; 2 Laboratory of Bioinformatics and Neurogenetics, Experimental Multiuser Laboratory (LaBiN/LEM), Graduate Program in Health Sciences, School of Medicine and Life Sciences, Pontifícia Universidade Católica do Paraná (PUCPR), Curitiba, Paraná, Brazil; 3 Hungarian Wrestling Academy “Kozma István”, Budapest, Hungary; 4 School of Doctoral Studies, Hungarian University of Sports Science, Budapest, Hungary; 5 Research Division, Buko Kaesemodel Institute (IBK), Curitiba, Paraná, Brazil; 6 Research Division, 9p Brazil Association (A9pB), Santa Maria, Rio Grande do Sul, Brazil; 7 Laboratory of Exercise Biochemistry in Health, Graduate Program in Health Sciences, School of Medicine, Pontifícia Universidade Católica do Paraná, Curitiba, Paraná, Brazil; 8 Faculty of Sport Sciences, Waseda University, Saitama, Japan; 9 Faculty of Sport Science, Ningbo University, Ningbo, China; 10 Institute of Sport Sciences and Physical Education, Faculty of Sciences, University of Pécs, Pécs, Hungary; 11 Department of Bioengineering, Sapientia Hungarian University of Transylvania, Miercurea Ciuc, Romania

**Keywords:** cardiovascular fitness, DMR (differentially methylated region), epigenome wide association study, EWAS, VO_2_max (maximal oxygen uptake)

## Abstract

**Introduction:**

Exercise is known to exert systemic effects associated with improved cardiovascular fitness and epigenetic modifications. However, the relationships between systemic epigenetic markers and physical fitness indicators remain insufficiently characterized.

**Methods:**

In the present study, the Illumina MethylationEPIC array (~730,000 CpG sites) was used to investigate associations between blood DNA methylation and key continuous fitness indicators-estimated VO₂max, grip strength, and vertical jump height-in a cohort of healthy adults. Epigenome-wide association analyses were adjusted for age, sex, estimated blood cell composition, and multiple testing.

**Results:**

No significant single-CpG associations were identified after rigorous adjustment. However, when participants were stratified into groups based on age- and sex-specific VO₂max norms, three CpG sites demonstrated differential methylation at a false discovery rate threshold (FDR < 0.05). These included cg21308111 in TNFRSF10A and cg17890932 in USP24, both showing higher methylation in lower-fitness groups, and an intergenic CpG site (cg02967877) exhibiting the opposite pattern. Region-based analyses further identified several differentially methylated regions (DMRs) between fitness groups.

**Discussion:**

These findings suggest that variation in cardiorespiratory fitness may be associated with subtle alterations in blood DNA methylation, particularly at loci related to metabolic regulation, inflammatory pathways, and intercellular communication. These findings shouldbe considered hypothesis-generating and require confirmation in larger, independent cohorts and, ideally, in longitudinal or intervention studies to elucidate more directly how changes in physical fitness relate to changes in DNA methylation patterns.

## 1 Introduction

Regular physical activity (RPA) confers broad, multi-system health benefits that extend well beyond the cardiovascular system and skeletal muscle. Habitual exercise improves vascular function and reduces arterial stiffness (Miyaki et al., 2009), and also enhances cerebrovascular compliance, thereby supporting improved brain perfusion (Barnes et al., 2021). These vascular and other systemic adaptations contribute to better cognitive performance: aerobic exercise promotes neuroplasticity and hippocampal function, boosting memory, learning, and pattern separation (Suwabe et al., 2017, 2018).). In addition, RPA exerts significant endocrine and metabolic effects - modulating levels of circulating hormones (Athanasiou et al., 2023), neurotrophic factors (Behrad et al., 2024), myokines (Pedersen, 2019), and inflammatory mediators (Nash et al., 2023) - and has been shown to influence gastrointestinal function, including gut microbiota composition (Torma et al., 2024). Notably, RPA is strongly associated with a reduced risk of numerous chronic diseases, including type 2 diabetes, obesity, several types of cancer, and neurodegenerative conditions such as Alzheimer’s disease (Radak et al., 2019). Moreover, meta-analytic data suggest a dose - response relationship between physical activity and all-cause mortality in middle-aged and older adults (Ekelund et al., 2019). Elite and high-performing athletic populations often demonstrate significantly increased life expectancy compared to their sedentary peers and even the general population (Kettunen et al., 2015; Runacres et al., 2021; Radak et al., 2025).

Recent lifestyle studies have linked greater physical fitness to a younger biological age. Individuals with higher fitness levels tend to exhibit a “younger” DNA methylation (DNAm)-based biological age, as reflected in epigenetic clocks such as GrimAge (Lu et al., 2019) and the more recently developed DNAmFitAge (McGreevy et al., 2023), along with a reduced risk of mortality. For instance, in the Rhineland cohort, RPA was associated with slower epigenetic aging even after adjusting for age, sex, smoking status, and cell proportion (Fox et al., 2023). In contrast, low levels of physical activity during aging may be accompanied by unfavourable epigenetic changes (You et al., 2025). These findings suggest that exercise and related lifestyle factors can influence the methylome in both muscle tissue and circulating cells, potentially mediating some of their systemic health effects.

Cardiorespiratory fitness, typically indexed by maximal oxygen uptake (VO_2_max or MET-level fitness), is an integrative phenotype that reflects not only the chronic history of physical activity and exercise training, but also genetic influences, early-life factors, comorbidities, and overall physiological reserve. Twin and family studies indicate that a substantial proportion of inter-individual variation in VO_2_max and in the response of VO_2_max to endurance training is heritable, underscoring that fitness at a given time point is not a simple proxy for recent exercise exposure alone (Schutte et al., 2016). At the same time, large cohort studies like such as the HERITAGE Family Study, have shown that the VO_2_max response to standardized endurance training also exhibits marked familial aggregation, with heritability estimates of approximately 40–50% for trainability (Bouchard et al., 1999). Thus, the level of cardiorespiratory fitness measured at a single time point cannot be interpreted as a pure proxy of recent exercise exposure, but arises from a combination of behavioural, biological and genetic determinants. Never the less cardiorespiratory fitness is a strong and independent predictor of morbidity and mortality, and in many analyses it is at least as strong, or stronger, than self-reported physical activity (Celis-Morales et al., 2017; Davidson et al., 2018). In such cohorts, individuals in the lowest fitness categories have markedly higher all-cause and cardiovascular mortality than those with moderate or high fitness, and each 1-MET increment in exercise capacity is associated with a meaningful reduction in mortality risk. These observations suggest that fitness level - regardless of whether it is determined predominantly by training or by other genetic and physiological factors - captures an aspect of physiological capacity that is highly relevant for long-term health.

Complementary fitness phenotypes such as handgrip strength and lower-limb power provide additional, partly distinct information on health and functional status. Lower grip strength is consistently associated with higher all-cause and cause-specific mortality and with greater risk of disability in community-dwelling adults (Wu et al., 2017; Lopez-Bueno et al., 2022), and it is increasingly proposed as a simple clinical marker of biological aging and frailty (Bohannon, 2019; Benton et al., 2022). Measures of explosive lower-limb power, including jump performance, have been linked to mobility limitation, gait speed and other functional outcomes in older adults (Reid and Fielding, 2012; Winger et al., 2021). Emerging evidence further suggests that lower-limb muscle power may be as important as - or even more important than - muscle strength for predicting functional capacity in this population (Van Roie et al., 2019).

Nevertheless, the amount of time spent engaging in physical activity does not necessarily translate directly into significant health benefits, and self-reported activity can be prone to recall and reporting bias (Talbot et al., 2002). To date, epigenome-wide association studies (EWAS) that analyse blood DNA methylation in relation to objectively measured physical fitness markers and apply thorough control for confounding factors remain scarce. Both DNA methylation and fitness phenotypes are influenced by age, sex, and leukocyte composition (as well as batch effects in the case of methylation measures), yet many prior reports did not always fully adjust for these factors. For example, peripheral blood methylation profiles can differ greatly simply due to shifts in white blood cell proportions, which must be accounted for to isolate fitness- or exercise-related signals. Moreover, fitness traits like VO_2_max and muscle strength also naturally vary by age and sex. In our previous study, we showed that biological age estimators are sensitive to different aspects of physical fitness (Jokai et al., 2023); however, there is limited information about individual methylation sites or regions associated with specific fitness phenotypes.

This gap in the literature, together with the need to better characterise the systemic signatures of physical fitness, motivated the present analysis. Here, we focus specifically on objectively measured fitness rather than self-reported exercise habits, and we interpret any associations as reflecting the current fitness status of the participants, recognising that this status is shaped by both exercise behaviour and underlying genetic and physiological factors. We aimed to test the hypothesis that robust and independent associations exist between whole-blood DNA methylation patterns and objective markers of physical fitness in humans.

## 2 Methods

### 2.1 Study population and physical fitness tests

Our study population consisted of 366 volunteers (192 females and 174 males) with a mean age of 60.5±10.3 years (see **Table 1** for details). A subset of the raw data, including DNA methylation RGChannelSet files, originally collected during the 2019 Masters Regatta Championship in Velence, Hungary (n=294), as previously described (Jokai et al., 2023), was included in the present analysis alongside novel data obtained on two subsequent occasions (n=72), during which participants visited our institute for testing and sample collection. All raw data - both previously collected and newly acquired - were processed and analysed using a novel analytical framework and an updated data processing pipeline, as described below.

**Table 1:** The basic characteristics of the study population.

Gender	N	Age (years)	Est. VO2 max (ml/kg/min)	Grip strength (kg)	Jump height (cm)	BMI (kg/m2)
Female	192	60.6 ± 9.7	36.5 ± 8.5	30.0 ± 5.7	23.9 ± 6.0	25.2 ± 3.4
Male	174	60.4 ± 11.0	42.4 ± 8.7	49.9 ± 9.1	31.3 ± 7.2	26.4 ± 3.4

Participants underwent the following sequence of assessments and sample collections. First, written informed consent was obtained, and the study procedures were explained in detail. A questionnaire was then administered to assess medical history of the participants, smoking and exercise habits. Smokers are excluded from the investigation because smoking has well-documented effects on cardiovascular and respiratory function, which could confound the assessment of relative aerobic capacity (Suminski et al., 2009) and blood based methlyation biomarkers (Lu et al., 2019). Other exclusion criteria included: acute or chronic musculoskeletal disorders, immunological diseases, a history of benign or malignant tumours, endocrine disorders, acute or chronic liver, kidney, or gastrointestinal diseases, a history of excessive alcohol consumption, mental health disorders, or blood coagulation problems.

Following the completion of anthropometric measurements (body mass and height), participants performed a maximal vertical jump test. The correct jumping technique was demonstrated by an instructor. A linear encoder was attached to a specialized belt fixed around the waist of the participant to measure jump height. After 1–2 practice jumps, three maximal effort jumps were recorded, and the best value was used in the statistical analysis (JumpMax[Cm]). Maximal grip force was measured using a hand dynamometer (expressed in kilograms: GripMax[kg]). Measurements were taken alternately from the left and right hands, and the highest value obtained was recorded as the maximal grip strength of the participant, as previously described (Jokai et al., 2023). Maximal relative aerobic capacity (VO2max) was estimated using the Chester Step Test, which assesses heart rate response across standardized exercise intensities (Buckley et al., 2004). Maximal heart rate was estimated using the Tanaka equation (Tanaka et al., 2001).

Participants were grouped into fitness categories based on their estimated VO2max, using previously published normative reference data (Kaminsky et al., 2015), stratified by age and sex. As shown by previous studies, individuals in the lowest quartile or quintile of age- and sex-specific exercise capacity are typically classified as having low fitness and have an approximately 2- to 5-fold higher risk of cardiovascular or all-cause mortality compared with those in higher fitness categories (Myers et al., 2002). Conversely, higher fitness levels, including very high cardiorespiratory fitness (e.g., ≥2 SD above the mean), have been associated with lower all-cause mortality compared with lower fitness groups (Mandsager et al., 2018). Accordingly, the Low-fit group included participants whose estimated VO2max was below the normative 25th percentile, while the High-fit group comprised those above the 75th percentile. Intermediate classifications were defined as Med-low-fit (0th–75th percentile) and Med-high-fit (25th–100th percentile), capturing participants between these extremes. The study protocol was approved by the National Public Health Center (approval number: 25167-6/2019/EÜIG) and was conducted in accordance with the ethical principles of the Declaration of Helsinki and applicable local national regulations.

### 2.2 Blood collection and procedures

Blood was collected 15–20 min before the physical fitness tests. Subjects were instructed to not to eat two hours before arriving to the examination as well as to avoid coffee or other caffeine contacting beverage consumption 12-hrs prior the procedures. Venous blood was collected from the cubital vein into EDTA containing vacutainer, frozen on dry ice and kept in -80 until subsequent DNA extraction and analysis.

### 2.3 DNA methylation measurement and bioinformatics

DNA mathlyation was measured on the llumina Infinium MethylationEPIC version1 BeadChip array (Illumina Inc., San Diego, CA, USA) as described previously (Jokai et al., 2023). Briefly, DNA was extracted from whole blood followed by bisulfite conversion. 500ng of high-quality genomic DNA (isolated by Pure LinkTM Genomic DNA Mini kit, Thermo Fisher, Carlsbad, CA, USA) per sample was processed using the EZ-96 DNA Methylation MagPrep Kit (Zymo Research, Irvine, CA, USA) in combination with the KingFisher Flex automated purification system (Thermo Fisher Scientific, Breda, Netherlands). To ensure unbiased sample processing, all samples were plated in a randomized order. During bisulfite treatment, 15μL of MagBinding Beads was used to facilitate DNA binding. The thermal conversion cycle included 16 repeated steps of denaturation at 95°C for 30 seconds, followed by incubation at 50°C for 60 minutes. After completion of the cycles, samples were held at 4°C for 10 minutes. Hybridization to the MethylationEPIC BeadChip array was performed using 8μL of the bisulfite-converted DNA as input material.

Raw DNA methylation data were obtained from peripheral blood samples using the Illumina Infinium MethylationEPIC BeadChip array and processed from IDAT files using the minfi version 1.52.1 (Aryee et al., 2014) and ChAMPversion 2.36.0 (Morris et al., 2014) packages within the R statistical environment. Standard quality control procedures were applied to ensure the reliability of the methylation measurements. Probes with detection p-values greater than 0.01 in more than 10% of samples, or with fewer than three beads in at least 10% of samples, were excluded. Additionally probes located on the X and Y chromosomes were excluded, as their methylation profiles reflect inherent chromosomal differences between sexes and do not allow for unbiased comparison.

To further improve the specificity of the data, we filtered out probes containing single-nucleotide polymorphisms (SNPs) at either the CpG site or the single-base extension, based on the annotation provided by Zhou et al (Zhou et al., 2017). Multi-hit probes that map to multiple genomic locations were also removed, following the probe list published by Nordlund et al (Nordlund et al., 2013). Signal intensities were preprocessed using the preprocessNoob() function from the minfi R package, which performs background correction and dye-bias normalization using the normal-exponential out-of-band (Noob) method.

After preprocessing and probe filtering, methylation levels at 735397 CpG sites were used for each participant in the form of a beta matrix. Beta values were logit-transformed into M-values for subsequent association analyses.

### 2.4 Epigenome-wide association of fitness markers and groups with DNA methylation sites

To account for cellular heterogeneity in whole blood samples, estimated cell-type proportions were derived using the Houseman method (Houseman et al., 2012), implemented within the minfi package using the estimateCellCounts2() function. For the estimation, we used the FlowSorted.Blood.EPIC reference library (Salas et al., 2018), which provides DNA methylation profiles of purified adult peripheral blood leukocyte subsets assayed on the Illumina HumanMethylationEPIC BeadArray. This reference panel includes CD8^+^ T cells, CD4^+^ T cells, natural killer (NK) cells, B cells, monocytes and granulocytes. No additional merging of cell types was performed. We inspected the distributions of the estimated cell fractions to confirm that values were within physiologically plausible ranges and that there were no extreme or truncated estimates, in order to ensure the quality of the deconvolution.

To further account for latent sources of technical or biological variation, principal components (PCs) derived from the methylation matrix were included as covariates using CpGassoc R package (Barfield et al., 2012). Only those PCs explaining more than 5% of the total variance were retained, as these were presumed to represent major non-random structure in the data. This results in the following model in the cpg.assoc() function: individual cpg site methylation is considered as dependent variable and Estimated VO2max, maximal Grip force, maximal Jump height or cardiovascular fitness categories are set as independent variable with the following covariates: biological sex, chronological age, blood cell composition variables, and PC explaining more than 5% of the total variance.

Power calculations were performed to assess the ability to detect single-CpG methylation effects. Under the assumption of a true underlying effect size of r= 0.35, adjusting for 10 covariates (age, sex, estimated cell composition, and principal components), and controlling for genome-wide multiple testing across 735,397 CpG sites using Holm/Bonferroni correction, a minimum of approximately 305 participants is required to achieve 80% power. Our study included 366 participants, providing sufficient power to detect moderate-to-large methylation effects at genome-wide significance. These calculations were based on the Fisher-Z transformation of partial correlations, accounting for the loss of degrees of freedom due to covariates.

### 2.5 Differential methylation region analysis of the different fitness groups and GO analysis

To identify differentially methylated regions (DMRs) associated with physical fitness phenotypes, the bumphunter function (version 1.48.0) from the minfi R/Bioconductor package (Jaffe et al., 2012; Aryee et al., 2014) was applied to M-values derived from the filtered and normalized data of the Illumina EPIC DNA methylation array. Analyses were conducted separately for two group comparisons: High-fit vs. Medium–Low-fit and Low-fit vs. Medium–High-fit individuals and with continuous markers of physical fitness. Prior to DMR detection, the methylation data were smoothed using the loessByCluster smoothing function to reduce technical noise and enhance regional signal detection.

The design matrix included covariates for chronological age, sex, estimated blood cell-type composition, and principal components as described above. A cutoff of 0.05 was applied to define candidate regions, and maxGap was set to 500 to limit the maximum genomic distance between neighbouring CpGs within the same candidate region. The statistical significance of each region was assessed using 1000 bootstrap permutations with nullMethod = “bootstrap”.

From the resulting gene sets of the differentially methylated regions, nominally significant (p<0.05) regions were mapped to the human genome using the GenomicRanges R package (version 1.58.0). The corresponding genes were then subjected to Gene Ontology (GO) analysis using the clusterProfiler R package (version 4.14.6) (Yu et al., 2012). P-values were adjusted using the Benjamini–Hochberg method to control the false discovery rate.

## 3 Results

### 3.1 Associations between blood DNA methylation and continuous markers physical fitness

**Figure 1:**
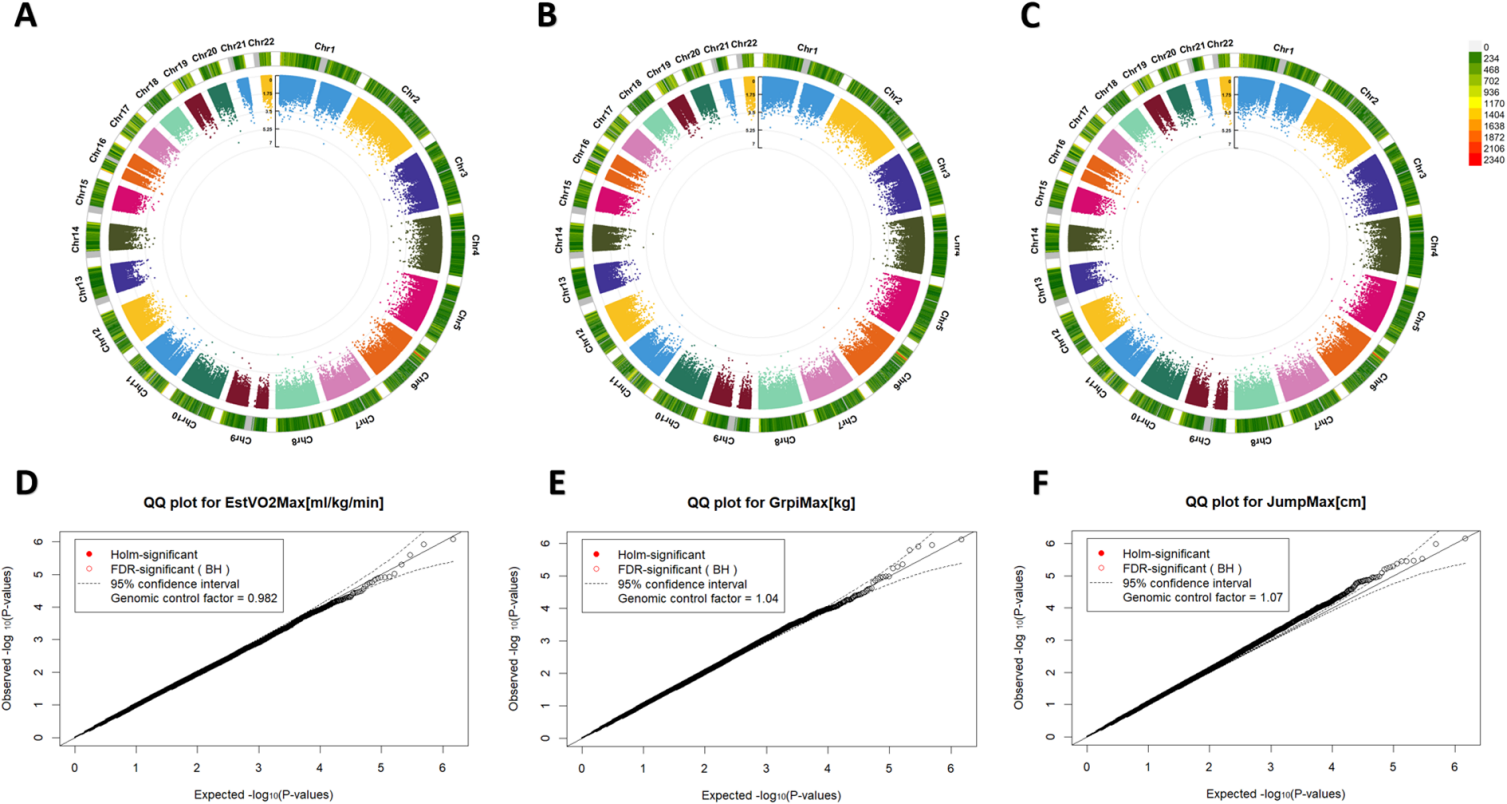
Epigenome-wide associations of continuous markers of physical fitness. **(A–C)** Circular Manhattan plots showing epigenome-wide association results across all human autosomes for estimated VO_2_max **(A)**, maximal grip force **(B)**, and maximal jump height **(C)**. The inner ring displays individual –log_10_(p-values), while the outer ring represents probe density, with colours corresponding to the scale shown in **(C)**. **(D–F)** Quantile–quantile (QQ) plots of –log_10_-transformed p-values from each EWAS for VO_2_max **(D)**, maximal grip force **(E)**, and maximal jump height **(F)**, respectively.

In the present study population, no significant associations were identified between DNA methylation levels measured using the Illumina EPIC array and key markers of physical fitness. Specifically, estimated VO2max (**Figures 1A, D**), maximal grip strength (**Figures 1B, E**) and vertical jump height (**Figures 1C, F**) showed no genome-wide significant CpG associations after rigorous adjustment for covariates. This held true under both Holm and Benjamini–Hochberg multiple testing correction thresholds.

### 3.2 DMS in low and high cardiovascular fitness groups

**Figure 2:**
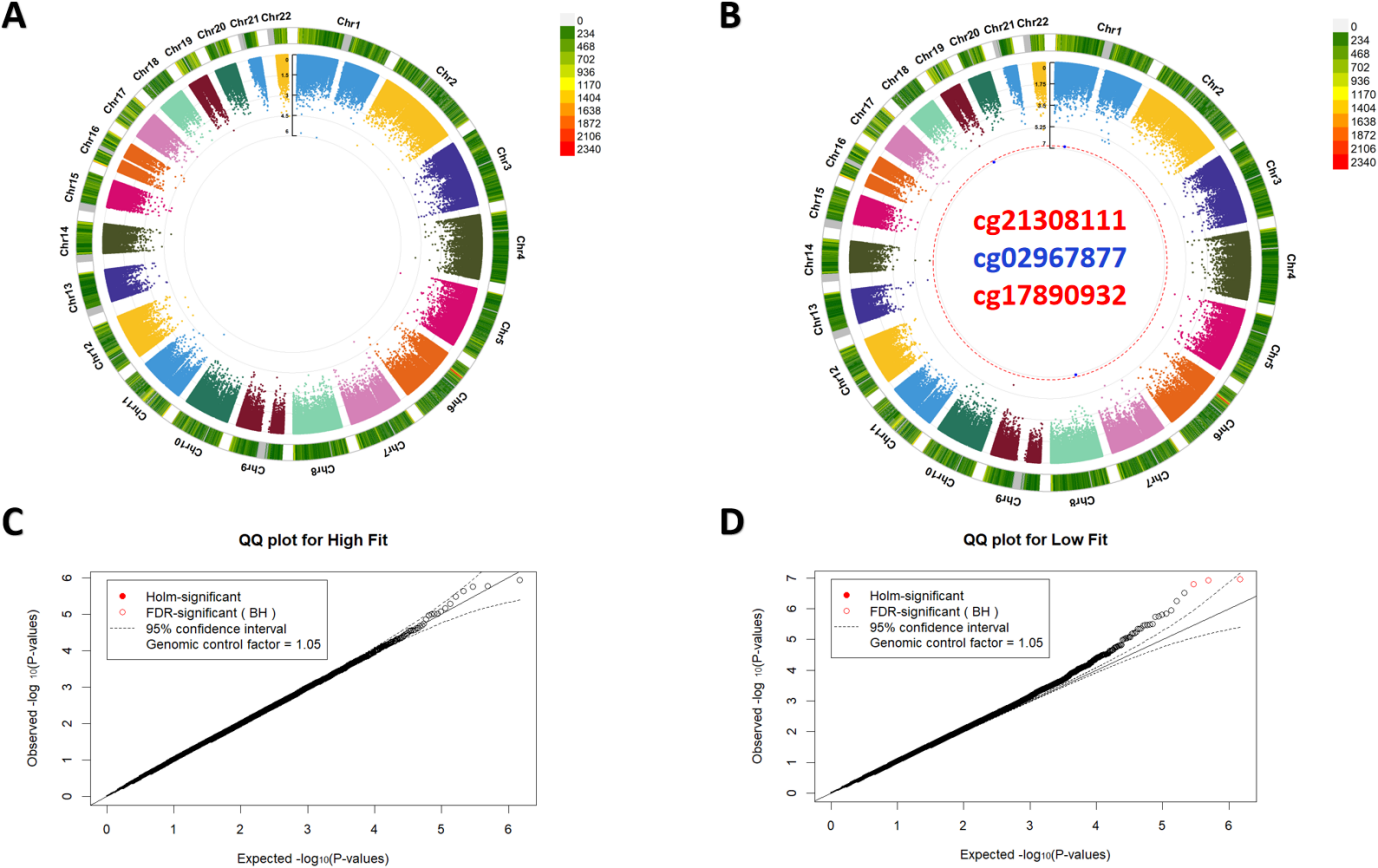
Differentially methylated probes in cardiovascular fitness groups. **(A, B)** Circular Manhattan plots showing the significance of differentially methylated sites (DMS) across all human autosomes for comparisons of High-fit vs. Med-low-fit **(A)** and Low-fit vs. Med-high-fit **(B)** groups. The inner ring displays individual –log_10_(p-values) for each probe, while the outer ring illustrates probe density, with colours corresponding to the scale shown on the side of the panels. The red dashed line indicates the Benjamini–Hochberg false discovery rate (FDR) significance threshold. **(C, D)** Quantile–quantile (QQ) plots of p-values from each comparison: High-fit vs. Med-low-fit **(C)** and Low-fit vs. Med-high-fit **(D)**, assessing deviation from the expected null distribution. Red open circles in panel D denote probes meeting the FDR significance threshold. In panel B, probe labels with red captions indicate positive associations with low-fit status, whereas blue captions indicate negative associations.

After finding no significant associations between continuous measures of physical fitness we investigated if there is difference in site specific DNA methlylation if our subjects are classified according to different cardiovascular fitness groups according to their gender and age group specific normative data. According to our data none of the measured probes methylation levels differ significantly between the High-fit and Med-low-fit groups (**Figures 2A, C**). On the other hand in case of Low-fit vs. Med-high-fit groups 3 probes cg21308111, cg02967877, cg17890932 shoved significant association at Benjamini–Hochberg multiple testing correction thresholds but not reached significance at the more strict Holm threshold (**Figures 2B, D**). cg21308111 was mapped to the TNFRSF10A gene (chromosome 8: 23,082,634), located within a CpG island. TNFRSF10A encodes a member of the tumour necrosis factor receptor super-family, with known roles in apoptotic signalling and inflammation. The site cg02967877, located on chromosome 19 (position: 37,800,260), falls outside annotated genes and has no currently assigned functional annotation. Lastly, cg17890932 was associated with USP24, a deubiquitinating enzyme, and is positioned in an open sea region on chromosome 1 (position: 55,675,110), potentially reflecting distal regulatory or intergenic activity. Among the FDR significant sites cg21308111 (t=5.42) and cg17890932 (t=5.35) showed positive association with low status of cardiovascular fitness and the intergenic region located cg02967877 (t=-5.4) had negative correlation after full covariate adjustment. Interestingly, when the data were analysed across age- and sex-stratified exercise group quartiles (Q1-Q4), no probes were significantly associated with cardiovascular fitness level, suggesting that the previously observed low-fit versus medium-high-fit association may reflect a contrast-specific group difference rather than a robust monotonic relationship across the full fitness distribution.

### 3.3 DMRs associated with fitness phenotypes and cardiovascular fitness stratification

From the continuous fitness markers only jump height produced candidate regional methylation signals. Three candidate regions were identified: one locus on chromosome 5 (chr5:139,076,600) overlapping the gene body of PCSK1; a second region on chromosome 10 (chr10:6,264,761–6,264,776) located in an intergenic region approximately 160 kb upstream of PRKCQ; and a third locus on chromosome 8 (chr8:143,751,796) located in an intergenic region approximately 180 kb upstream of MROH6. However, none of these candidate regions reached even nominal statistical significance. In case of Low-fit groups compared to Med-high-fit individuals out of the 8370 “bumps”, 675 region with functional annotation was nominally significant (**Supplementary Table 1**). Between the High-fit and Med-low-fit groups from the 3060 hits only 227 annotated gene was nominally significant (**Supplementary Table 2**). The top 10 deferentially methylated regions is shown on the **Table 2**.

**Table 2:** Top 10 deferentially methylated regions in different groups of cardiovascular fitness.

Top 10 differentially methylated regions									
Low-fit vs. Med-High-fit groups									
Chr	Start	End	Value	Area	L	Cluster L	P value	FWER	Gene
chr5	178986131	178986906	-0.51403724	7.196521	14	14	6.60E-06	0.047	GRM6
chr12	130821607	130822818	-0.37791490	4.157064	11	14	5.62E-05	0.346	STX2
chr20	36148604	36149656	-0.08362898	2.676127	32	43	1.28E-04	0.630	EPB41L1
chr21	36258423	36259797	-0.25828509	3.357706	13	13	1.78E-04	0.752	DOP1B
chr4	124232	125504	0.34593886	2.767511	8	8	1.82E-04	0.745	ZNF718
chr8	57358130	57360727	0.08668316	1.993713	23	27	2.45E-04	0.825	LOC101929488
chr11	61594708	61596812	0.08805995	1.937319	22	22	2.66E-04	0.854	LOC101927495
chr7	38350464	38351468	0.33065002	2.314550	7	7	2.77E-04	0.875	TRG-AS1
chr1	248099880	248101009	-0.28238968	2.541507	9	12	2.91E-04	0.874	OR2L13
chr2	198650112	198651498	-0.11721401	2.109852	18	29	3.08E-04	0.936	LOC105373831
High-fit vs. Med-Low-fit groups									
Chr	Start	End	Value	Area	L	Cluster L	P value	FWER	Gene
chr19	2282568	2282928	-0.62053225	1.8615967	3	8	1.36E-05	0.041	SPPL2B
chr6	291687	293285	-0.34405159	3.4405159	10	10	5.78E-05	0.165	DUSP22
chr20	36148457	36149750	0.08706575	3.0473013	35	43	7.77E-05	0.218	EPB41L1
chr5	178986131	178986906	0.24707526	3.4590537	14	14	8.96E-05	0.251	GRM6
chr15	90198832	90198832	-0.40590933	0.4059093	1	9	3.25E-04	0.624	SEMA4B
chr18	74961724	74962794	-0.07609309	1.3696757	18	34	6.87E-04	0.911	ZNF407
chr2	3704363	3705271	-0.20951431	1.6761145	8	9	7.43E-04	0.895	DCDC2C
chr11	5617367	5618408	-0.20348944	1.6279156	8	10	8.29E-04	0.918	TRIM5
chr4	124232	125504	0.19802799	1.5842239	8	8	9.24E-04	0.937	ZNF718
chr7	766100	766384	0.21013711	1.2608227	6	19	1.12E-03	0.962	DNAAF5

Differentially methylated regions were ranked by region-level significance in two pairwise contrasts of cardiovascular fitness groups. The upper panel lists the top 10 DMRs for Low-fit vs. Med-high-fit, and the lower panel for High-fit vs. Med-low-fit.

Each column in the table is defined as follows. Chr denotes the chromosome on which the differentially methylated region (DMR) is located. Start and End specify the genomic start and end coordinates (hg19 assembly) of the DMR, respectively. Value represents the average difference in smoothed methylation between the two groups. Area is the region-level test statistic, calculated as the sum of t-statistics across all probes within the DMR. L indicates the number of probes comprising the initial candidate region, while ClusterL gives the number of probes after merging adjacent candidate regions into a single DMR. p-value are the nominal p-value obtained from the regional permutation test and FWER is the family-wise error rate across all tested regions. Gene column lists the nearest or overlapping gene annotation for each DMR.

### 3.4 Gene ontology analysis of DMR-s

**Figure 3:**
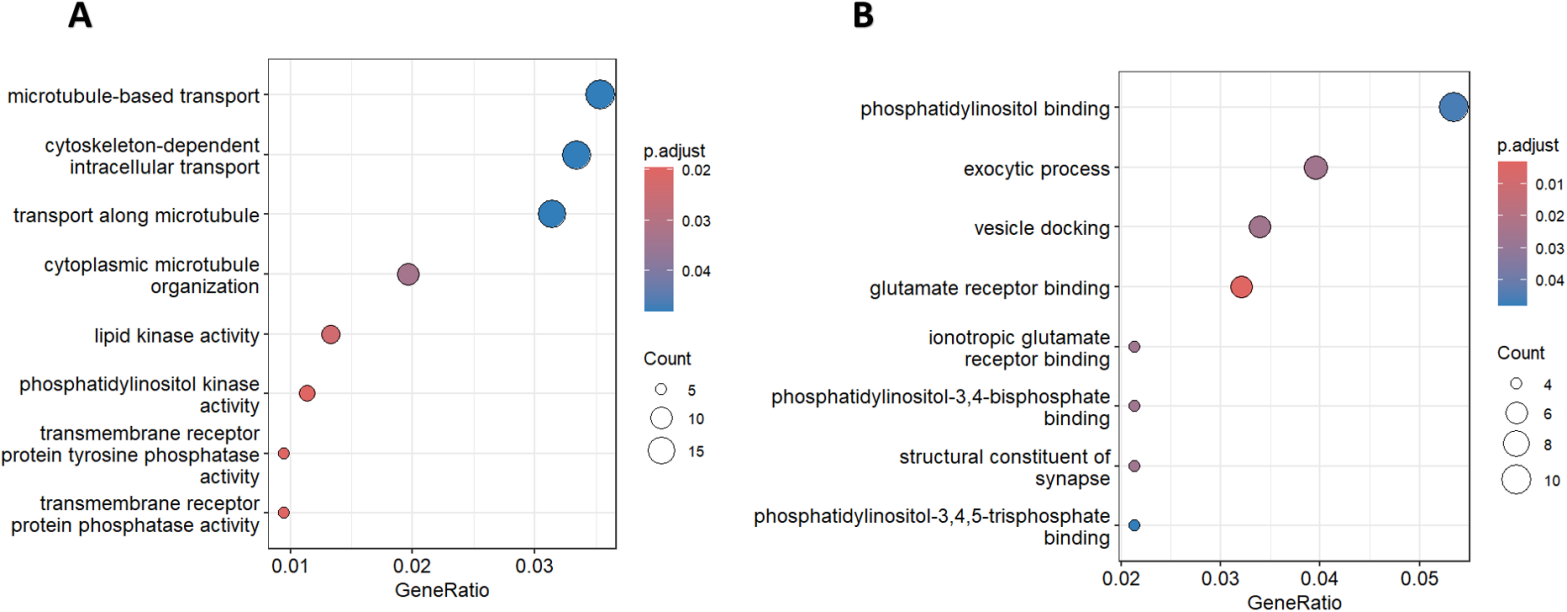
Gene ontology analysis in different groups of cardiovascular fitness. **(A)** Significant GO terms for differentially methylated regions (DMRs) between Low-fit and Med-high-fit groups. **(B)** Significant GO terms for DMRs between High-fit and Med-low-fit groups. The size of each dot indicates the number of genes involved, and the heat chart represents the level of significance after FDR adjustment.

Our exploratory gene ontology analysis revealed that differentially methylated regions in low-fit individuals were significantly associated with microtubule-based transport, cytoskeleton-dependent intracellular trafficking, cytoplasmic microtubule organization, lipid kinase activity, phosphatidylinositol-related processes, and transmembrane receptor protein phosphatase activity (**Figure 3A**). In contrast, high-fit individuals showed enrichment in pathways related to phosphatidylinositol binding, exocytic processes, vesicle docking, glutamate receptor binding, ionotropic glutamate receptor binding, and binding to phosphatidylinositol 3,4-bisphosphate and phosphatidylinositol 3,4,5-trisphosphate (**Figure 3B**).

## 4 Discussion

In the present epigenetic investigation of physical fitness, we did not detect genome-wide significant associations between blood DNA methylation (~730,000 CpG sites) and key fitness measures (estimated VO_2_max, grip strength, vertical jump height) after covariate adjustment and correction for multiple testing. In other words, baseline fitness levels were not accompanied by large, robust differences at individual CpG sites in blood in this cohort. Similar observations have been reported previously. In Chinese monozygotic twins, only 25 CpGs in 9 genes, including FBLN1, RXRA, and ABHD14B, were associated with grip strength (Luo et al., 2024). Likewise, our findings resemble those of Soerensen and colleagues (Soerensen et al., 2019) who examined the relationship between handgrip strength and blood DNA methylation in more than 600 middle-aged and older monozygotic twins and did not identify CpG sites reaching epigenome-wide significance after adjustment for sex, age, and blood cell composition. Their suggestive findings at the regional level pointed to immunological and cell differentiation pathways. Taken together, these studies support the view that any associations between physical fitness and the blood methylome are likely to be modest and may require very large samples or alternative analytical strategies to detect reliably.

Concomitantly, a recent meta-analysis suggests that VO_2_max shows a more pronounced, although still moderate, association with skeletal muscle DNA methylation patterns (Voisin et al., 2024). At the same time, regular exercise does not necessarily translate into proportional changes in fitness parameters, and epigenetic signatures of habitual activity appear subtle: in a meta-analysis of ~900 individuals, only nine CpGs were associated with accelerometer-measured physical activity at p < 1×10^-5^, and just one site (cg07030336 in VTI1A) replicated in an independent cohort (Nishida et al., 2022). That study reported higher physical activity to be associated with higher methylation at VTI1A, a vesicle-trafficking gene, with the CpG also related to inflammatory markers. Despite substantial advances in exercise epigenetics, it remains uncertain whether, and through which mechanisms, exercise-induced tissue-specific methylation changes causally influence fitness traits such as VO_2_max, strength, or power, or whether they predominantly mirror pre-existing differences between individuals. Because these fitness parameters are key determinants of functional capacity and health, clarifying how exercise-related methylation changes manifest across different tissues will require well-powered longitudinal intervention studies with repeated epigenetic, gene expression and fitness assessments.

Although we did not identify genome-wide significant differentially methylated sites associated with continuous measures of physical fitness, we performed secondary, exploratory analyses by comparing individuals at opposite sides of the fitness distribution to those with intermediate levels. Participants were stratified into High-fit, Low-fit, Med-low-fit, and Med-high-fit groups based on age- and sex-specific VO_2_max norms (Kaminsky et al., 2015).). No single CpG reached significance after Holm correction in comparisons between fitness groups (High vs. Med-low and Low vs. Med-high). However, using a false discovery rate (Benjamini–Hochberg, FDR 5%) as a more permissive criterion, three CpG sites were flagged in the Low vs. Med-high comparison.

Two of these loci map to genes with plausible roles in stress and inflammatory processes. TNFRSF10A (TRAIL receptor 1; probe cg21308111) encodes a member of the tumour necrosis factor receptor superfamily involved in apoptotic signalling and inflammation, with cg21308111 located in a CpG island within the gene (Elias et al., 2009; Kaczynski et al., 2023) USP24 (cg17890932, open-sea region on chr1) encodes a deubiquitinating enzyme implicated in the regulation of protein turnover and stress responses, including DNA damage signalling via p53 stabilization (Zhang and Gong, 2016). The third site, cg02967877, is intergenic (chr19:37,800,260) and currently lacks functional annotation. In our data, lower fitness status was associated with higher methylation at the TNFRSF10A and USP24 sites (positive t-values for Low-fit status), whereas cg02967877 showed the opposite pattern (lower methylation in Low-fit or higher in Med-high-fit). These effect sizes were modest, and the findings should be considered exploratory, but they are compatible with the hypothesis that lower cardiorespiratory fitness may coincide with subtle epigenetic differences in genes linked to inflammation/apoptosis (TNFRSF10A) and cellular stress regulation (USP24).

Recent work further underscores the potential relevance of these genes in immune and inflammatory contexts. USP24 has been implicated in reduced antitumor activity in T-cells via a STAT3-related pathway (Hsieh et al., 2025), and other studies suggest that USP24 is responsive to chronic inflammatory environments, contributing to phenotypes such as intervertebral disc degeneration (Li et al., 2023) and tumour progression *In vitro* experiments indicate that overexpression of nuclear factor kappa B (NF-κB) and tumour necrosis factor alpha (TNF-α) increases USP24 promoter activity. TNF-α shares upstream signalling features with DR4 (Death Receptor 4, encoded by TNFRSF10A) and TRAIL (TNF-related apoptosis-inducing ligand), both of which are involved in immune surveillance, tumour suppression, and resolution of inflammation (Wang and El-Deiry, 2003). While our cross-sectional data do not allow mechanistic inference, the involvement of TNFRSF10A- and USP24-related sites fits within a broader inflammatory and stress-response framework.

Pathway-level analyses offered additional insights. Gene ontology enrichment in High-fit individuals highlighted phosphatidylinositol binding and signalling, closely related to insulin action via the phosphoinositide 3-kinase (PI3K)–Akt pathway, a central intracellular cascade activated by insulin. This pathway was enriched among differentially methylated regions (DMRs) in the High-fit group and may be consistent with more favourable glycaemic homeostasis and insulin sensitivity - outcomes often associated with regular physical activity (Wu et al., 2018). A recent study also emphasizes the role of circulating systemic factors: in a rat model, administration of plasma from young animals to aged recipients induced differential DNA methylation enriched in insulin-related and immune system pathways (Chiavellini et al., 2024) suggesting that blood-borne signals can modulate epigenetic states in a way that relates to metabolic regulation.

The PI3K-related pathway enrichment we observed in both Low-fit and High-fit DMRs aligns with known metabolic differences across fitness strata. The PI3K–Akt pathway is essential for insulin action, glucose uptake, and lipid metabolism. Low cardiorespiratory fitness is frequently accompanied by higher visceral adiposity (Rheaume et al., 2011; Obirikorang et al., 2018), insulin resistance, and dyslipidaemia (Carnethon et al., 2003; Erez et al., 2015). Conversely, higher cardiovascular fitness has been associated with improved insulin sensitivity (Haufe et al., 2010) and more favourable lipid profiles (reduced triglycerides and LDL-cholesterol, increased HDL-cholesterol) (Alghadir et al., 2024). In our GO analysis, Low-fit individuals showed regional methylation differences in genes with phosphatidylinositol kinase activity, whereas High-fit individuals showed differences in phosphatidylinositol-binding and downstream signalling. Although these findings require replication, they are consistent with the idea that endurance-trained individuals may exhibit distinct epigenetic regulation of insulin/Akt-related genes as part of an adaptive response to regular exercise. Supporting this notion, a 14-week combined aerobic and resistance training intervention in middle-aged women induced significant DNA methylation changes in more than 800 blood CpG sites, enriched in metabolic pathways including AMPK, TGF-β, and insulin signalling, and was accompanied by improved fitness and reduced blood pressure and triglycerides (da Silva Rodrigues et al., 2023). In that trial, key genes in insulin signalling pathways became hypermethylated, suggesting a possible link between epigenetic modifications and improved metabolic health. While our cross-sectional analysis did not include direct measures of insulin resistance or lipid levels, the PI3K- and lipid-kinase–related pathways identified in Low-fit individuals could plausibly relate to higher metabolic risk, a hypothesis that warrants direct testing in future work.

Low fitness, inflammation, and insulin resistance are interrelated. TNF-α, which is elevated in conditions linked to sedentary lifestyles (Alzamil, 2020), can impair insulin signalling and promote insulin resistance, whereas regular exercise can reduce TNF-α and help preserve insulin sensitivity (Olson et al., 2012; Alzamil, 2020). Thus, the epigenetic signals observed here - TNF-related CpG sites and PI3K pathway genes - may represent interconnected components of the broader network linking cardiovascular fitness to metabolic health, though the directionality and causality of these relationships remain to be established.

It is also notable that some pathways highlighted in our analysis - such as cytoskeletal organization, microtubule-based transport, and glutamate receptor binding - are consistent with the multisystem nature of exercise adaptation. Regular physical activity enhances not only metabolic and immune function but also neural control and neuromuscular communication, which may leave epigenetic traces in circulating cells. The enrichment of glutamate receptor binding among High-fit DMRs is of interest in this respect. Although glutamate is best known as a central excitatory neurotransmitter involved in synaptic plasticity (Kennedy, 2013), peripheral immune cells can also express glutamate receptors (Pacheco et al., 2007; Sadat-Shirazi et al., 2018). Thus, glutamate receptor-related methylation differences in blood may reflect local immune signalling processes rather than direct neurogenic changes. Alternatively, they may indicate broader neuroimmune or neuroendocrine influences of sustained physical fitness, potentially mediated by brain-derived or hormonal signals, although this remains speculative and requires targeted investigation.

In our High-fit group, DMR-related gene sets were also enriched in GO terms related to exocytic processes and vesicle docking, suggesting a potential involvement of extracellular vesicle-mediated signalling. In previous work, we found that individuals with high cardiovascular fitness displayed distinct microvesicle cargo profiles (Gyorgy et al., 2025), which related to the DNAmFitAge estimator (McGreevy et al., 2023). Horowitz et al. demonstrated that plasma from exercised mice improved hippocampus-dependent learning and memory and increased DCX+ cell counts, indicative of adult hippocampal neurogenesis (Horowitz et al., 2020). More recently, intravenous administration of genetically engineered senescence-resistant human mesenchymal progenitor cells ameliorated age-associated phenotypes in multiple organ systems in animal models, with part of the benefit attributed to exosomal processes (Lei et al., 2025).

Overall, these observations are compatible with the idea that systemic changes are not merely secondary to organ-specific adaptations but may themselves influence organ function, potentially creating a positive feedback loop. Our findings align with and extend prior reports, including the epigenome-wide study by Duncan et al (Duncan et al., 2022), which showed that habitual physical activity is associated with systemic epigenetic differences detectable in buccal cells of monozygotic twins. The authors concluded that physical activity is linked to epigenetic patterns favouring a healthier metabolic profile. In our cohort, cardiovascular fitness status was associated at nominal significance levels with differential DNA methylation patterns in circulating blood, independent of age, sex, and estimated cell composition. Notably, in participants with high cardiovascular fitness, we observed enrichment signals in extracellular vesicle-related pathways - an aspect not highlighted in the twin study, which primarily focused on metabolic traits such as BMI and waist circumference. While these observations require cautious interpretation, they raise the possibility that high cardiorespiratory fitness is accompanied by specific epigenetic adaptations related to intercellular communication and systemic resilience.

Taken together, our results are consistent with an emerging framework in which regular exercise and higher physical fitness are linked to systemic biological changes relevant for healthy aging. We acknowledge that our cross-sectional fitness measures capture more than prior exercise exposure alone (e.g., genetic, developmental, and other lifestyle influences). Nonetheless, from an epigenetic perspective, exercise remains a particularly important and modifiable determinant, with the potential to alter fitness-related traits and broader health outcomes, possibly even lifespan. Epigenetic regulation – including DNA methylation – is a plausible contributor, as it can integrate environmental influences such as physical activity over time. Exercise has been shown to induce tissue-specific methylation changes; for example, long-term training in skeletal muscle affects genes involved in structural remodelling, energy metabolism, and mitochondrial function (Geiger et al., 2024), while short-term training in mice alters methylation at genes related to muscle growth, differentiation, and metabolic regulation (Kanzleiter et al., 2015). Our findings add blood-based evidence that aligns with these broader patterns, but further mechanistic and longitudinal work will be necessary to disentangle causal pathways and the specific contribution of exercise-related versus other influences on physical fitness status.

Our study has several limitations that should be acknowledged. First, the cross-sectional design provides only a snapshot of physical fitness and methylation at a single time point, precluding conclusions about directionality or causality. Second, we were unable to confirm our findings in an independent dataset, as, to our knowledge at the time of writing, there are no publicly available large-scale datasets that simultaneously include the specific cardiovascular fitness markers examined here and genome-wide blood methylation profiles. Third, we applied a relatively lenient threshold for defining differentially methylated regions (p < 0.05 without FWER correction), prioritizing sensitivity over strict control of type I error. This was an intentional strategy to enable gene ontology enrichment on a broader set of loci and should be viewed as hypothesis-generating rather than definitive. Within this context, the top-ranked DMRs and enriched GO terms indicate biologically plausible connections to fitness-related pathways (e.g., insulin signalling) and point to potential roles for inter-organ communication, including exosomal processes, in the systemic effects of regular physical activity. These hypotheses will need to be tested and refined in larger, longitudinal, and mechanistically oriented studies.

## 5 Conclusion

In summary, this epigenome-wide analysis did not provide strong evidence for single-CpG DNA methylation associations with blood-based markers of physical fitness (estimated VO_2_max, grip strength, and vertical jump height) after adjustment for age, sex, and estimated cell type composition. In exploratory group comparisons, Low-fit individuals showed suggestive methylation differences at loci related to apoptosis (TNFRSF10A) and proteostasis (USP24). Comparisons across the fitness strata identified these subtle differences at loci linked to inflammatory and ubiquitination pathways, as well as small regional changes. At the pathway level, these signals implicated processes such as intracellular transport, phosphatidylinositol/PI3K–Akt signalling, and extracellular vesicle–related mechanisms. Confirmation of these exploratory findings in larger, independent cohorts - and ideally in longitudinal or intervention studies with repeated measures of both fitness and DNA methylation across multiple tissues as well as blood - will be essential to clarify the nature and directionality of fitness–epigenetic relationships.

Taken together, our findings are compatible with the notion that physical fitness is accompanied by a diffuse epigenetic footprint in blood, rather than by large, easily detectable shifts at individual CpG sites. They also fit with the well-established physiological links between fitness, inflammation, and metabolic regulation, but suggest that the epigenetic connections between fitness measures and systemic versus tissue-specific adaptations are likely to be complex and distributed across multiple pathways.

## Data Availability

The datasets generated during the current study are available in the Gene Expression Omnibus (GEO) repository, with accession number: GSE305174: https://www.ncbi.nlm.nih.gov/geo/query/acc.cgi?acc=GSE305174.
